# Uncontrolled Bleeding After Tongue Laceration Leading to a Difficult Airway in the Setting of Hemophilia A: A Case Report

**DOI:** 10.7759/cureus.31455

**Published:** 2022-11-13

**Authors:** Rebecca Siegel, Dalal Budri, John Morrison

**Affiliations:** 1 Department of Emergency Medicine, Brookdale University Hospital and Medical Center, Brooklyn, USA; 2 Department of Emergency Medicine, Centinela Hospital Medical Center, Inglewood, USA

**Keywords:** difficult airway management, hemophilia a, intubation in sitting position, difficult hemostasis, airway bleeding

## Abstract

Bleeding in the upper airway is a potentially catastrophic event and an important cause of airway-related mortality, even in otherwise healthy patients. The bleeding airway is particularly challenging because tools used by physicians to secure a difficult airway, such as direct video and flexible video/fiberoptic laryngoscopy, may be ineffective as the camera may become soiled with blood. A 33-year-old male with hemophilia A presented with a tongue laceration after a seizure. We present an upper airway emergency due to hemorrhage in hemophilia A, effectively managed with a strategy employing pre-intubation optimization, semi-recumbent position, and video laryngoscopy.

## Introduction

Bleeding in the upper airway is a potentially catastrophic event and a common cause of airway-related mortality, even in otherwise healthy patients [1]. The bleeding airway is particularly challenging because tools that are traditionally used by physicians to secure a difficult airway, such as direct video and flexible video laryngoscopy, may be ineffective as the camera may become soiled with blood [2,3]. In a retrospective, intensive care unit-based study that looked at the reason for past pass intubation failure with direct video laryngoscopy, blood in the airway was one of the top three reasons for failed attempts at intubation (the other two were cervical immobility and edema in the airway) [2]. The challenge of securing a bleeding airway becomes even more important in the context of a patient with coagulopathies. Hemophilia A, which is the most common hereditary disorder of hemostasis, occurs in one out of every 5,000 males and accounts for 80% of hemophilia cases [4]. Patients with hemophilia A have a 13% chance of some airway disorder involving oropharyngeal bleeding, with an 8% chance that it will be immediately life-threatening [5]. Here, we describe a case of a 33-year-old male with hemophilia A who presented to the emergency department (ED) with a seizure that resulted in a tongue laceration with difficult-to-control bleeding. The patient ultimately required intubation for airway protection when hemostasis could not be achieved.

## Case presentation

A 33-year-old male with a history of seizure disorder, alcohol use, and hemophilia A came to the ED with a chief complaint of seizure. The patient had a brief seizure and subsequent postictal state lasting 10 minutes, returning to mental baseline by the time of arrival at the ED. Initial vitals were blood pressure of 96/79, heart rate of 99 beats per minute, and respiratory rate of 15 breaths per minute. A head computed tomography scan was performed, which was unremarkable. The patient was observed to be mentating at baseline and without deficit for several hours prior to discharge. Shortly after the patient's discharge, the patient had another seizure in which he sustained a tongue laceration. Physical examination revealed a significant amount of blood oozing from the mouth. The patient was given 2 g of intravenous levetiracetam as well as 2,000 units of recombinant human factor VII. Indications for factor VII were active bleeding with potential airway compromise and an elevated prothrombin time of 79.1. Despite initial hemostasis with direct pressure, the patient continued to have intractable bleeding, requiring hemostasis with lidocaine, epinephrine, and suture. The patient tolerated this poorly and was noted to have progressive lethargy and difficulty clearing his airway between hemostasis attempts, the last of which caused desaturation and airway compromise.

Given the patient’s difficulty clearing his airway and the risk of impending airway compromise, the decision was made to intubate the patient. In anticipation of a difficulty, in which a cricothyrotomy would likely exacerbate the patient’s bleeding, it was important to secure the airway on the first pass. The patient was pre-oxygenated for 10 minutes in the upright position (position maintained with assistance from the bed and members of the team) with a nasal cannula and face mask, which were intermittently removed for suctioning. A timeout was called, during which the emergency medicine team discussed potential airway strategies. On the pre-intubation assessment, the patient was noted to have developed a large, roughly 3 x 3 x 2 cm, non-hemostatic clot on the dorsum of the tongue overlying the laceration. Dual suction was prepared in anticipation of this clot becoming displaced and subsequent worsening hemorrhage. The team desired a fiberoptic nasal approach; however, this was unavailable at the time. In lieu of that, hyperangulated video laryngoscopy, using the GlideScope (Verathon Inc., Bothell, Washington, United States), was selected over regular video laryngoscopy or direct visualization due to resident comfort and blade angulation, allowing for a “backup” or semi-recumbent approach. Supine intubation with a standard geometry video laryngoscopy and a bougie was selected for backup, due to its ability to convert to a direct approach if soiled. Rapid sequence intubation was used with etomidate as an induction agent and rocuronium as a paralytic. The patient was lowered from 90 degrees to semi-recumbent at ~45 degrees, which aided in hemorrhage control. Initial insertion of the blade disrupted the clot as anticipated; however, care was taken to clear the clot with the distal part of the blade on insertion, resulting in the camera being distal to bleed, which allowed for a grade I view of the vocal cords, which is the most complete view and includes full visualization of the glottis. The patient’s position and dual suction controlled bleeding, allowing for a window during which the team had first-pass success. The patient was admitted and successfully extubated two days later. The patient’s total hospital stay was five days.

## Discussion

Tongue lacerations are a relatively common injury seen in the ED and can commonly result from a variety of means including seizures, self-harm, blunt force facial trauma, oral trauma while intubated, and child abuse [6]. While hemostasis can usually be easily achieved with direct pressure, this may not always be the case, particularly in patients with lingual artery lacerations [7] and patients with coagulopathies such as hemophilia A.

The mainstay of treatment in hemophilia A, both in prophylaxis and in the actively bleeding patient, is the administration of systemic factor VIII; though in places where concentrated factor VIII is not available, the administration of cryoprecipitate and fresh frozen plasma may be suitable alternatives [8]. In hemophilia A patients who are actively bleeding, desmopressin and tranexamic acid have been shown to be helpful adjuncts in a variety of cases [8]. Topical hemostats, such as local fibrin glue and chitosan-based dressings, have been shown to be efficacious in controlling hemorrhage in hemophilia A patients undergoing surgery [9], though their usefulness in primary hemorrhage has not been adequately studied.

While techniques for managing a difficult airway in hemophilia A patients may be similar to the generalized difficult airway approach in patients with an expanding hematoma, in cases of actively hemorrhaging upper airway, video-based methods of intubation such as fiberoptic and direct video laryngoscopy intubation may be less helpful, meriting a new approach. A 2019 review on managing and securing the bleeding upper airway outlines many approaches to this predicament [1]. The article emphasizes pre-oxygenation, limiting the bleeding, and reversal of underlying coagulopathy as initial steps and then instructs physicians to keep the patient in an upright posture while attempting to visualize vocal cords and turning to a “backup” method if they are unable to be visualized. The article describes that until the patient is unconscious, they need to be in a sitting position to avoid choking on blood but that once the patient is unconscious, the patient can be placed in a sniffing or head-down position based on circumstance and provider preference. Backup methods detailed in this article include supraglottic airway devices (only suitable if bleeding is above the larynx), oral digital intubation, blind nasal intubation, retrograde intubation, and ultrasound-guided intubation [1].

## Conclusions

Here, we have reported a case of uncontrolled bleeding from a tongue laceration, requiring intubation in the setting of hemophilia A. While tongue lacerations are a common finding in a patient who has had a seizure, special considerations must be made in those patients who have coagulation disorders, as these patients present a unique risk of airway compromise. While this risk is unique in the severity and management of hemorrhage, it is also unique in the stark absence of good options for a surgical airway. Due to this, in addition to the medical and surgical management of these patients, an early plan for securing the airway is vital. A comprehensive intubation strategy with contingency should be tailored individually to each patient's presentation. In our case report, we found that a video-assisted, upright approach, with dual suction, allowed for first-pass success.
